# Supplementary material for: Category-specific perceptual learning of robust object recognition modelled using deep neural networks
Source: PLoS Comput Biol. 2025 Sep 23;21(9):e1013529. doi: 10.1371/journal.pcbi.1013529 (PMC12478885; doi:10.1371/journal.pcbi.1013529)
Supplement: Attachment — Submitted filename: Jang_NoisePercLearn_2024_ResponseLetter_v4.pdf [file pcbi.1013529.s005.pdf]

REVIEWER COMMENTS

Reviewer's Responses to Questions

Reviewer #1: Two experiments explored the relationship between object recognition robustness and training on images of objects that were degraded by Gaussian noise. This was done for both human subjects and several well-studied CNN models to determine whether CNNs can be used to model the perceptual learning occurring in humans. In Experiment 1, the researchers found that both humans and CNNs showed significant benefits (lower thresholds) from noise training. These benefits were larger for the models, but the authors showed that this was due to the pretrained models having zero experience with noisy images (unlike humans). In Experiment 2, the researchers asked a similar question only now manipulated whether the category of the object was animate or inanimate to determine whether the training benefits are category general or category specific. They found that, for humans, there were training benefits only for the trained-on category (category-specific), whereas CNNs showed both category-general and category-specific learning. The researchers also showed that, once CNNs were pre-trained with noisy images, they too showed only category-specific training benefits (like humans). From layer analyses of the CNNs, they concluded that category-general learning is due to weight changes in the lower layers whereas category-specific learning is due to weight changes in the higher layers.

I appreciated the opportunity to read this paper. The research question is interesting, important, and as the authors state, relatively unstudied. The authors fill this gap with a solid study that answers some basic questions about the relationship between object recognition robustness and training on noise-degraded images. The data patterns are clear and well analyzed, and the conclusions do not overstep the results of these analyses. My only criticisms in reading the manuscript were in wanting the see the two very things that the researchers mention in future work (lines 376-386). Greedily, I would have liked to see these extensions in the current manuscript, but neither extension is easy and publication of the current work shouldn't be delayed in the interim. When the authors do get around to these directions, they should consider the ImageNet-C dataset (Hendrycks D, Dietterich TG. Benchmarking Neural Network Robustness to Common Corruptions and Perturbations. ICLR 2019) that introduces a range of visual corruptions to ImageNet images. With respect to their goal of considering the role of recurrency in training, they may want to consider a recent paper that used recurrency to model top-down processing and showed it to improve object recognition robustness (Ahn, S., Adeli, H., & Zelinsky, G. J. The attentive reconstruction of objects facilitates robust object recognition. PLOS Computational Biology, 20(6) 2024).

We greatly appreciate the reviewer's positive comments about this work and the helpful suggestions provided. Below we address the individual points raised.

First, thanks for pointing out the recent study by Ahn et al. The modeling results are of considerable interest and relevance, and our revised general discussion section describes this recent work, namely how adding a reconstructive attentional mechanism to a DNN model led to improved robustness to stimuli presented in visual noise.

We agree that expanding the analysis of object recognition robustness under various visual corruptions is a crucial avenue for further research. In this regard, one of our previous studies investigated how training with blurred images enhances generalizability to other types of noise and improves neural predictivity (Jang & Tong, Nature Communications, 2024). That study also provided an analysis showing improved robustness from blur training on the ImageNet-C benchmark.

Encouraged by Reviewer 1's suggestions, we have performed an additional analysis on all of our

DNNs trained on Gaussian-noise-degraded animate or inanimate objects from 16 categories, using the ImageNet-C benchmark (Hendrycks & Dietterich, 2019). The results of this new analysis, which are now included as **Figure 6** in the revised manuscript, and are also presented below.

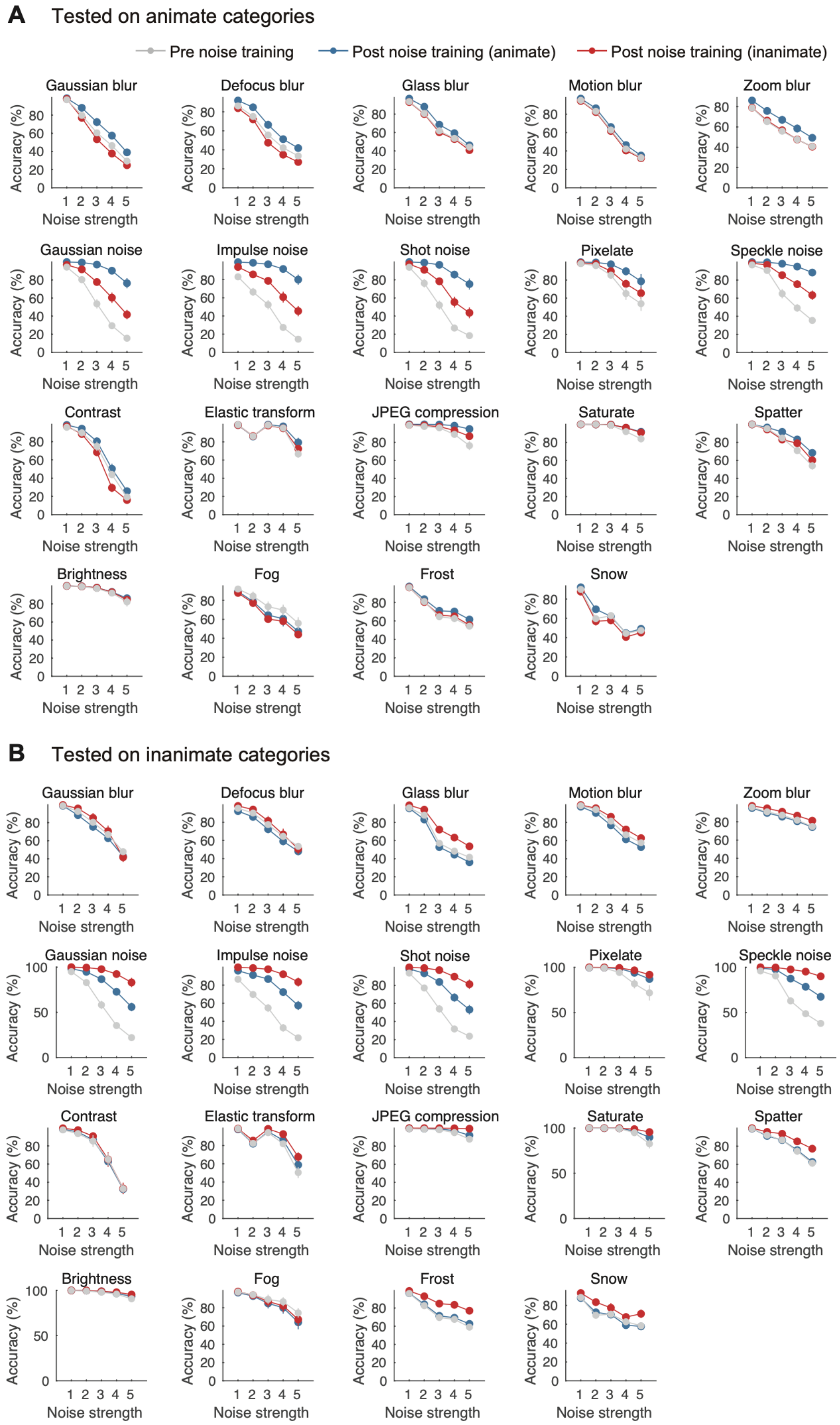

This new analysis reveals that the noise-trained CNNs demonstrate strong generalization to multiple types of noise that somewhat resemble Gaussian noise, including impulse noise, shot noise, and speckle noise, as well as certain other types of image degradation (e.g., pixelate, JPEG

compression). This contrasts with the findings of Geirhos et al. (2018), who reported extremely poor generalization when networks were tested on other distortion types (e.g., from salt-and-pepper noise to uniform noise, or vice versa). There were also suggestive trends of better performance for certain types of blur for the trained category, but these effects were relatively small and varied across animate and inanimate object categories.

Additionally, within the Gaussian noise family, category-specific and category-general learning effects remained evident, with networks trained on animate objects outperforming those trained on inanimate objects when tested on animate categories, and vice versa. However, this pattern did not extend beyond Gaussian noise. We have added a discussion of these new analyses to the revised Results and also to the revised Discussion.

Perhaps one small way that the manuscript could be strengthened is if a stronger connection were made to the attention literature. Humans get constant training on noisy images in the context of normal active vision and the blur/noise that is experienced in peripheral vision. An object selected for fixation is first perceived as blurred in the visual periphery, but the visual system then gets immediate feedback on its high-resolution version following the eye movement to that object. This is an even more specific form of noise training than in the researchers' category-specific condition because the noise is embedded in the specific object to be recognized, not an object that is categorically related. It would be interesting to know how this object-specific training benefit would compare to the category-specific and category-general benefits reported here. My intuition is that this sort of training benefit would be captured by weights in the lower layers, but this would seem to break with the pattern reported here by the researchers, where category-specific benefits are associated with learning in the higher layers. If the researchers choose to make this connection in their introduction, they may want to look at studies that predict the movements of a foveated retina (e.g., Yang, Z., Mondal, S., Ahn, S., Xue, R., Zelinsky, G., Hoai, M., & Samaras, D. Unifying Top-down and Bottom-up Scanpath Prediction Using Transformers. CVPR 2024; Yang, Z., Huang, L., Chen, Y., Wei, Z., Ahn, S., Zelinsky, G., … & Hoai, M. Predicting Goal-directed Human Attention Using Inverse Reinforcement Learning. CVPR 2020), which is a related question.

It is true that attention to the periphery and eye movements could have an important role in object learning. That said, our study was not designed to address this question. Eye movements were not monitored in our study, and our pre- and post-test measures of SSNR thresholds did not allow for eye movements. Also, the DNNs we worked with processed each object image in its entirety and did not involve simulated eye movements. For these reasons, we have opted not to discuss issues pertaining to foveation in the current study.

Some small things.

Line 89. "fundamentally misaligned" seems a bit strong. Augmenting CNN training with noisy images easily fixed this problem, so it couldn't be fundamental.

The wording here has been revised to state that CNNs trained exclusively with clear images "sharply deviate from how the human brain processes visual information".

Lines 93-94. This suggestion of a biological circuit sounded to me a bit of a straw man. If a simple denoising circuit exists in the visual system, wouldn't someone have found it by now? Is there evidence for such a circuit? And if there was a de-noising circuit, shouldn't there be minimal to no perceptual learning, which is known not to be true? My point being, a data-driven approach based on perceptual learning and experience just seems far more plausible.

In our view, the notion that the visual system might have some inherent properties that allow it to be somewhat robust to noise (either external or internal) is a fairly longstanding idea, though not universally discussed. We have modified the text here to include the term "filtering", before denoising and deblurring, to broaden the message here, as even a simple filter such as a difference of Gaussian filter (like retinal ganglion cells) or a Gabor filter (like a V1 neuron) can serve to improve robustness to pixelated noise to some extent. We have also added some references (Atick & Redlich, Neural Computation, 1990; Lu & Dosher, Psychological Review, 2008) relevant to this point.

Line 233. Figure 3D is referenced in the text but is missing from the caption.

Thank you for catching this oversight. We have corrected this, and **Figure 3D** is now accurately referenced in both the text and the figure caption.

Reviewer #2: In this paper, the authors measured human behavioral performance and DNN output to understand how training on noise image input would affect object recognition performance. They found in both humans and DNNs that training with noisy images improves performance on novel images not included in training and that training may affect performance in a category-specific manner.

Overall, I found the study to be cleverly designed and well conducted, and the results are clean and informative. The contrast between human and DNN performance is particularly informative and allows us to gain a deeper understanding of how training on noisy images may affect object recognition performance. I just have one comment that I would like the authors to consider.

The authors argue that training is category-specific. However, I wonder if training is, in fact, object-specific. To show that training is truly category-specific, training should only involve half of the animate or inanimate objects. If the effect of training is generalized to the untrained objects in the same animate or inanimate category, then we have support for the category specificity of training. The results as they stand right now, however, are also consistent with object-specific training: once the human observer or DNN learns to recognize a particular object in noise, they can generalize such learning to new exemplars of the same object; if training only involved, e.g., animate objects, then performance would only improve for those objects and not inanimate objects. This possibility should be addressed, perhaps with a new study. I believe that addressing this point thoroughly will make this study a more significant contribution to the field.

We thank the reviewer for raising this important point, which was also raised by Reviewer 3. These suggestions motivated us to add a new paragraph to the general discussion to address these issues in greater detail. First, to ensure that we are using a common terminology, the classic studies by Rosch et al., 1976 would describe the 16-category classification task that we employed to be at the basic level, while the distinction between animate and inanimate categories would be at the superordinate level. Our study focused on perceptual learning for superordinate-level categories, as it is well documented that animate objects (in our case, animals and mostly mammals) tend to share similar intermediate-level visual properties (e.g., curvy rather than rectilinear, fur-like texture in many cases, medial axis body structure plus extensions, etc.). A similar case can be made for inanimate manmade objects, which consist of intermediate visual features that are quite distinct from those of animate objects (e.g., Long, Yu & Konkle, PNAS, 2018). Thus, by testing the effects of perceptual learning for more visually distinct superordinate categories (i.e., animate vs. inanimate), we could maximize the likelihood of detecting category-specific effects if it were the case that perceptual training did indeed lead to specificity of learning in our human observers.

While this approach allowed us to test for category-specific effects of learning at the superordinate level, it leaves open the question of whether training with one set of basic-level categories, say belonging to the animate superordinate level, would lead to complete, partial or no generalization to other untrained animate categories. We openly discuss these issues in our revised discussion section, and indicate that this would constitute an interesting question for future research but falls outside of the goals of the current study. Our revised discussion makes clear that we find positive evidence of category-specific learning at the superordinate level, and is careful not to overstate the scope of the conclusions that can be made from our positive empirical findings.

Reviewer #3: This paper presents a novel perspective on noise robustness in human object recognition compared to artificial neural networks (ANNs) pretrained on image classification. The study makes two key contributions: First, it demonstrates that humans' robust perception of noisy images partially stems from their exposure to visual noise during development. The authors show that pretraining ANNs on noisy natural images improves their robustness in object categorization. This suggests that the previously observed gap in robustness between humans and ANNs can be, at least partially, attributed to differences in training exposure. Second, they demonstrate that additional training with noisy images leads to category-specific improvements that don't generalize to other categories - a pattern observed in both humans and ANNs. This kind of side-by-side comparison of human and ANN learning processes offers insights into the strengths and limitations of neural network models while advancing our theoretical understanding of neural and behavioral aspects of learning in humans and other animals. For these reasons, and also the importance of presented results, I strongly support the publication of this paper. However, I have a few concerns that should be addressed:

We appreciate the reviewer's positive feedback and their comments on finding our presented results to be of importance. Below, we address each of the specific concerns raised in detail.

Major Comments: Choice of animate vs. inanimate Categories: While the brain shows distinct structural differences in processing animate versus inanimate objects, I'm not aware of similar systematic differences in ImageNet-trained ANNs. The authors should either provide evidence for this distinction in ANNs or justify their category choice. Why not use finer-grained object categories to demonstrate category-specific robustness? For instance, training on two object categories and testing robustness on a third? Does the degree of category-specificity depend on the choice of train/test categories? Empirical evidence showing the phenomenon's consistency across different category combinations would strengthen the findings.

Based on the reviewer's input, we have modified the introduction section to clarify that our rationale for comparing animate versus inanimate categories was motivated by the fact that these categories are known to differ in their mid-level visual properties (e.g., Long, Yu & Konkle, PNAS, 2018) and to activate differentiated representations in the human ventral visual system (e.g., Kriegeskorte et al., 2008; Sha, et al., 2015; Bracci & Op de Beeck, 2016; Jozwik & Kriegeskorte, 2022; Coggan & Tong, 2023; Contier et al., 2024). Thus, if training were to induce perceptually specific changes in the visual system, corresponding to the properties of the trained objects rather than the background noise, then one should be better positioned to detect category-specific effects of learning if those categories share less overlap in their visual representations. For these reasons, we chose to evaluate the effects of perceptual learning at this broader level.

In the modified discussion, we point out that our study tested for category-specific effects at this

broader level, and we indicate the conclusions that can be drawn from these positive empirical findings.

As we also noted in our response to Reviewer 2, the idea of testing for specificity or partial transfer of learning across visually similar basic level categories (say consisting different animal categories) would be an interesting to pursue in future research, and would constitute a separate new study in its own right. Such a study would likely require training and testing a much larger number of participants to achieve sufficient statistical power to test for the possible presence of weaker effects of learning (i.e., effects of partial generalization). To provide relevant context, Experiment 1 of this manuscript required 1 semester of data collection to complete (20 participants x 4 sessions or 80 experimental sessions), and Experiment 2 required 1.5 semester to complete (32 participants x 3 sessions or 96 experimental sessions).

Regarding whether DNNs show a similar animate-inanimate distinction as the human visual system, research suggests that supervised CNN models better capture these tendencies than simpler models, though probably not to the same extent as the primate visual system (e.g., Khaligh-Razavi & Kriegeskorte, 2014). The focus of our study was on the acquisition of robustness to visual noise, not on issues of the representational distance between many possible object categories, so this issue, while interesting in its own right, does not directly bear on the main findings of our study.

Layer-specific effects: The authors should consider an ablation study to support their claims about layer-related effects on category-general and category-specific robustness. For example, freezing early layers during fine-tuning could verify whether this prevents category-general improvements.

We thank the reviewer for this thoughtful suggestion. Motivated by these comments, we implemented an additional analysis to explore the impact freezing the higher or lower layers of VGG-19, followed by training with noisy animate or noisy inanimate objects. It should be noted that these analyses are not meant to be exhaustive, which could constitute a major study in its own right, but rather, to provide a supporting line of evidence to the layer-specific noise sensitivity analysis that was provided with the original submitted manuscript (i.e., **Figure 4**).

As can be seen in the figure below, when learning was restricted to the early layers (i.e., layers 1-4, with layers 5-19 frozen), we primarily observed category-general effects of training with noisy objects. By contrast, when learning was restricted to the higher layers (i.e., layers 13-19, with layers 1-12 frozen), we primarily observed category-specific effects of learning. It should also be added that clear images from the "untrained" category were concurrently provided (with matched frequency) as part of our training procedures. These findings provide further evidence to support our interpretation that category-general acquisition of noise robustness relies on earlier stages of visual representation while category-specific learning of robustness relies on higher levels of visual representation. Based on these positive supporting findings, we have added a new figure (**Figure 5**) that displays these new analysis results.

As a side note, we found that to obtain category-general effects of DNN training, it was necessary to freeze the intermediate and higher layers of VGG-19, as considerable category-specific information resides in the intermediate layers of VGG-19. This side note is mentioned in the caption for **Figure 5**, in case a few readers are interested in such specifics.

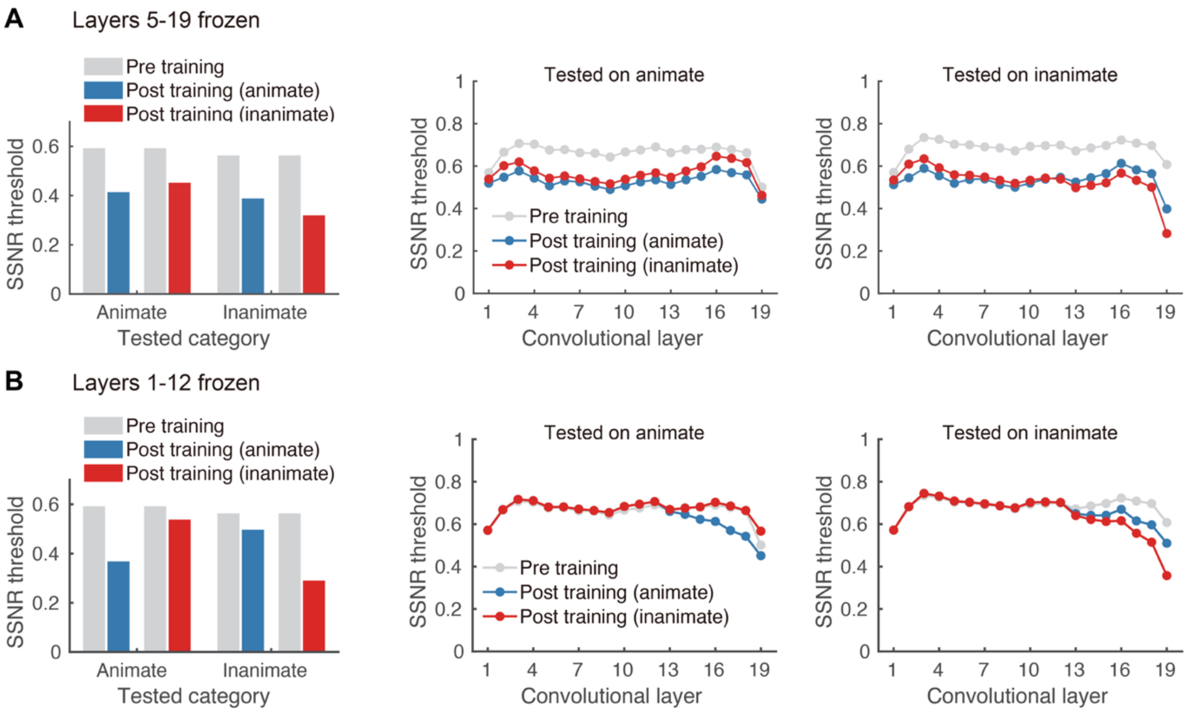

Impact on noiseless image performance: How does training with noisy samples affect the model's performance on standard ImageNet images? Are there meaningful changes in classification accuracy?

In our previously published work (Jang et al., PLOS Biology, 2021), we found that DNNs needed to be trained on a mixture of noisy and noise-free object images, otherwise performance for clear images would start to decline severely due to overspecialization and catastrophic forgetting. In the current study, noisy object images were presented with noise levels randomly drawn from a uniform distribution ranging from 0.2 to 1.0, so fairly clear images were presented quite often.

We have added a new plot to **Figure 2** (see also below) to show mean classification accuracy as a function of SSNR level for the noise-trained DNNs from experiment 1. This shows the improved performance for noisy objects across a wide range of SSNR levels. There is also a very small drop in performance at high SSNR levels, but this had minimal impact on our estimates of SSNR thresholds (based on 50% accuracy for 16 alternative classification in Experiment 1).

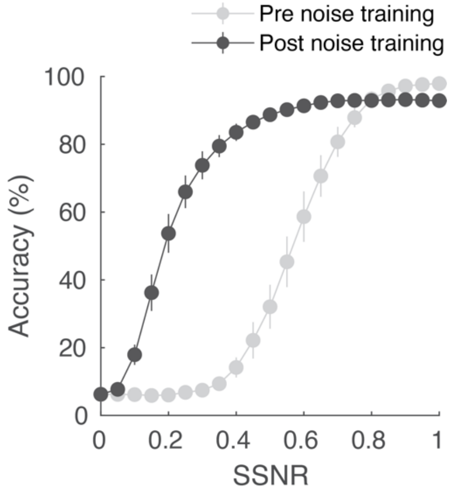

Generalization of noise robustness: While exposure to noise during training may enhance robustness, it may not be the only contributing factor. Does training with Gaussian noise, as used in this study, improve robustness to other types of noise? How do early layer modifications affect performance on clean images? Do these layers respond similarly to different types of noise?

The reviewer raises an important question about whether training with Gaussian noise generalizes to other noise types. Based on this query and also Reviewer 1's query, we evaluated our pre-trained and noise-trained DNN models with the ImageNet-C benchmark (Hendrycks & Dietterich, 2019), which evaluates robustness to many different image perturbations including multiple forms of noise and blur. The results can be seen in our response to Reviewer 1, and have been added as **Figure 6** in our revised manuscript.

Our results showed that these networks generalized well to noise types closely related to Gaussian noise (e.g., impulse, shot, and speckle), and showed a modest incremental benefit on certain other types of image degradation (e.g., pixelated, JPEG compression). In related work, we have shown that DNNs trained with a mixture of blurry and clear images show considerable improvements in robustness to both blur and noise (Jang & Tong, 2024).

The question regarding the role of the early layers was addressed above.

Minor Comments:
Please clarify how the choice of data augmentation techniques during model training influenced the robustness results.

We have added to the Methods section that we applied only minimal data augmentation techniques, specifically RandomResizedCrop and RandomHorizontalFlip. We avoided using methods such as color jitter, which would be quite similar to adding noise to the images.

For the SSNR threshold learning curves in Figures 2 and 3, please specify whether these represent individual or averaged subject data. What is the inter-subject variability? Does the observed effect (category-specific robustness) appear in individual subjects or only in group-level analyses?

For the SSNR threshold learning curves in **Figures 2** and **3**, the plotted values represent the data averaged across all individual participants. The adaptive staircase procedure allows one to obtain threshold estimates from each participant for each condition. The shaded regions indicating ±1 standard error of the mean as a measure of inter-subject variability. We have clarified this point in the figure captions of the revised manuscript.

Because animate and inanimate categories were presented randomly during training, it is challenging to disentangle category-specific from category-general effects solely through these learning curves. Consequently, the bar graphs of category-specific and category-general effects were derived from the Quest procedure data collected both before and after the training sessions, providing a more direct assessment of these distinctions.
