# Supplementary material for: Category-specific perceptual learning of robust object recognition modelled using deep neural networks
Source: PLoS Comput Biol. 2025 Sep 23;21(9):e1013529. doi: 10.1371/journal.pcbi.1013529 (PMC12478885; doi:10.1371/journal.pcbi.1013529)
Supplement: Attachment — Submitted filename: Jang_NoisePercLearn_2024_ResponseLetter4_Final.pdf [file pcbi.1013529.s006.pdf]

REVIEWER COMMENTS

Reviewer's Responses to Questions

Reviewer #1: The researchers satisfied all of my concerns.

Reviewer #2: I am not sure the authors convincingly addressed my comment. To argue that the training effect is specific to the animate or inanimate distinction at the superordinate category level, it would be necessary to test animate and inanimate subordinate categories that are not included in the training. One could, for example, train on bear, bison, elephant, hamster, hare, and test on lion, owl, tabby cat. If training on animate subordinate categories improves performance on the untrained animate subordinate categories, but not on the untrained inanimate subordinate categories, then we can conclude with confidence that training indeed improves performance at the superordinate category level. Without doing so, I am not sure it is valid to make such a claim. I am not trying to be difficult here, and I would like to be convinced. It should be easy to at least present some CNN results with such a manipulation?

We appreciate the reviewer's feedback and suggestions, and note that Reviewer 3 made a similar recommendation. First, we would like to clarify that our intention was not to assert that the training effect was at the level of the animate vs. inanimate superordinate categories, as we did not make any assertions in the manuscript text as to whether training with a subset of animate (or inanimate) categories should or should not lead to generalization to other untrained categories from the same superordinate category. Rather, our choice of experimental design was to enhance the ability to detect potential category-specific effects of learning, should they exist, and we chose the animate/inanimate distinction because it is known to produce well-differentiated representations in the human ventral visual cortex and in DNNs. Since animate and inanimate object categories are known to differ in their intermediate-level visual properties (e.g., Long et al., *PNAS*, 2018), such differences could also potentially support category-specific effects of perceptual learning.

That said, we agree with the reviewer that testing the generalization of DNNs to untrained basic categories from the trained superordinate class is addressable and could provide some insight into the degree of specificity or generality that takes place with DNN training. Based on these recommendations, we conducted a new set of DNN experiments in which separate sets of DNNs were trained with one of four distinct groups of categories: Animate1 (bison, hamster, lion, owl), Animate2 (bear, elephant, cat, hare), Inanimate1 (airliner, jeep, speedboat, table lamp), or Inanimate2 (sports car, couch, schooner, teapot). This design allowed us to test whether training might lead to successful generalization to untrained basic-level categories from the same superordinate category.

From the figure shown below, it can be seen that training with subsets of categories led to category-specific improvements at the basic level in 3 out of 4 cases, while we also observed a significant improvement in SSNR thresholds for the DNNs that were trained with Animate 1 and subsequently tested with Animate 2 ($t(5) = 2.72$, $p = 0.0418$). These new analysis results indicate that DNNs trained with noisy object images predominantly exhibit improvements for those trained categories at the basic level, with only limited generalization observed within superordinate domains. Based on Reviewer 2's recommendations, we have added a short description of these new analysis results to the revised general discussion section, and have included the figure shown here as **Fig S2** of the revised manuscript.

Animate: Bison, Hamster, Lion, Owl, Bear, Hare, Cat, Elephant
Inanimate: Airliner, Jeep, Speedboat, Table lamp, Couch, Sports car, Schooner, Teapot
Animate1: Bison, Hamster, Lion, Owl
Animate2: Bear, Hare, Cat, Elephant
Inanimate1: Airliner, Jeep, Speedboat, Table lamp
Inanimate2: Couch, Sports car, Schooner, Teapot

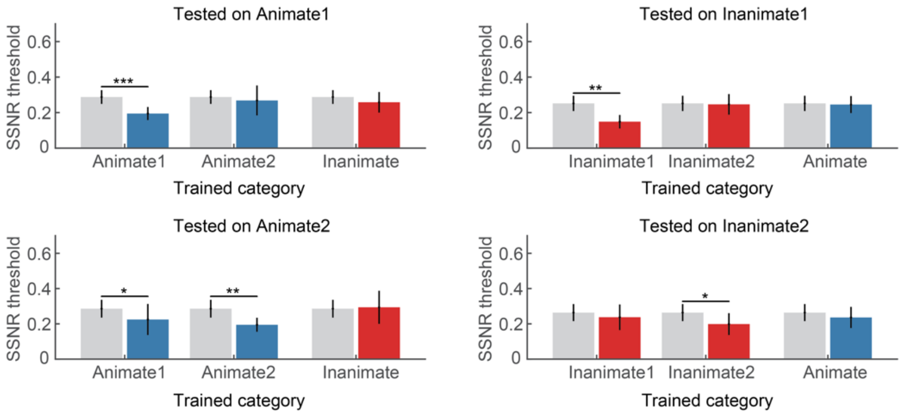

Reviewer #3: I thank the authors for their engagement with my previous comments and for providing new analyses. While some concerns have been addressed, I still have a few important issues that need to be addressed:

1. Regarding category-specificity, I remain unconvinced by the authors' response. Given that the paper makes general claims about "category specificity" rather than just "animate vs. inanimate category specificity," I believe it's reasonable and within scope to demonstrate category specificity for at least a few additional categories in the ANN models. While collecting new human data would be outside the scope of the current work, testing other categories in the computational models should be feasible. If the authors disagree, I request a more substantial justification.

We thank the reviewer for the suggestions, and note that Reviewer #2 raised similar concerns about the nature and scope of category specificity for the trained DNN models. In response, we have conducted a new series of DNN experiments designed to directly test generalization across untrained categories within both animate and inanimate superordinate classes. These analyses, now detailed in our response to Reviewer #2 and included in the revised manuscript (**Fig S2**), systematically evaluate the extent to which training effects generalize to other basic-level categories from the same superordinate category.

The results indicate clear improvements in robustness for categories that were included in training, reflecting strong basic-level category specificity. We also observed limited generalization to untrained categories within the same superordinate class, though these effects were less consistent and observed in just 1 of 4 of the sets of analyses (i.e., when training on Animate1 and testing on Animate2). With these additional analyses now included in the revised manuscript, we believe our study has been considerably strengthened and extended as a result of the review process.

2. Regarding the layer freezing experiment conducted in response to my previous comment, the selection of layers appears somewhat arbitrary: layers 1-12 (13 layers) were frozen as "early" layers, while layers 5-19 (15 layers) were frozen as "deep" layers. These ranges, in my view, are too extensive to be meaningfully categorized as either "deep" or "early." Each experiment freezes approximately 70-75% of the network's layers. Could the authors clarify their rationale for determining

which layers to freeze? Additionally, when intermediate and deep layers are frozen (layers 5-19), we observe category specificity in the SSNR threshold of deep layers that mirrors the pattern seen when freezing layers 1-12, though with relatively smaller magnitude. The two panels in the third column of the new Figure 5 display very similar patterns in the deep layers. This suggests that the differential change in layers' SSNR cannot fully explain the category specificity, since the same differential pattern emerges in deep layers even when they're frozen. This requires further explanation. Since this freezing experiment is the only reliable evidence for the layer-dependency of the category specificity, I believe it needs to be conducted exhaustively and clearly explained.

The reviewer points out highly relevant issues, and it is evident from the questions raised that we were not sufficiently clear in our previous submission at explaining the factors that motivated us to select relevant layers for the freezing manipulations. To clarify, the layer ranges used in our initial freezing experiments were not selected arbitrarily, but rather, were motivated by the pattern of layer-wise SSNR thresholds found in **Fig 4**. We have revised the results section to make the following points clearer. In **Fig 4** of the manuscript, it can be seen that category-general benefits of noisy object training increased considerably (relative to pre-training performance) across lower layers 1-4, with these benefits sustained through the intermediate layers (approximately layers 5-12), after which a sharp divergence in SSNR thresholds can be observed in layers 13 and above for trained versus untrained categories. These empirical findings motivated us to investigate the consequences of only allowing the lowest layers (1 to 4) to be trainable due to the category-general gains in noise robustness observed there, or only the higher layers (13 to 19) to be trainable due to the category-specific changes observed in those layers.

Nonetheless, we agree with the reviewer that it would be more informative to show the effects of freezing a smaller portion of the lower or higher layers to illustrate the consequences of allowing the effects of training to extend into the intermediate layers. To this end, we have added a supplementary figure (**Fig S1**) to the manuscript which shows the effects of freezing a varying number of the intermediate and higher layers (i.e., layers 5-19, 9-19, 13-19, or 17-19 frozen) while leaving the layers below trainable. In a complementary set of analyses, we froze a varying number of the lower layers (layers 1-16, 1-12, 1-8, or 1-4) so that training could take place in a variable number of the layers above. The results of these analyses are shown below.

As expected, the most extreme differences in terms of category-general or category-specific patterns of change in SSNR thresholds is observed in the top row, where the majority of the higher layers (left) or lower layers (right) were frozen, respectively. This is evident in the classification-based SSNR thresholds (i.e., bar plots) and can also be seen in layer-wise correlation-based measures of SSNR thresholds. The reviewer is correct that for the layer-wise analysis, there remains a small difference between trained and untrained categories in the higher layers when only layers 1-4 are trainable (left top plot). With the additional analyses shown, it can be seen that when additional trainable layers are included (e.g., layers 1-8, second row, left), the divergence between trained and untrained categories becomes magnified considerably. As we note in the revised results section, these findings are consistent with the fact that differences between animate and inanimate visual categories are known to emerge at intermediate levels of CNN models (e.g., Long et al., *PNAS*, 2018). Thus, when these networks are set to have both lower and intermediate layers trainable, and are then trained with noisy animate (or inanimate) objects, these intermediate representations can become modified to become more noise robust. What is interesting and somewhat counterintuitive at first glance is that these differences are observed downstream from the site of training, with these correlation-based SSNR measures diverging in the higher frozen layers. It should be kept in mind that the training-induced loss function is still to improve noise robustness for the trained stimuli at the final classification stage (with the error signal backpropagated through the network) so this will necessarily entail changes in how the higher frozen layers respond to the trained stimuli (and possibly also untrained stimuli) as a

consequence of training, based on the series of feedforward operations that must take place for the signals to reach the higher layers.

It may also be worth noting that the pattern of results seen on left plots, say with layers 1-8 trainable, differs somewhat from when all layers are trainable, as SSNR thresholds do not continue to decrease in the higher frozen layers, as they do in the entirely trainable version of VGG19 (**Fig 4A**), so noise robustness does not continue to improve in the higher frozen layers. Another trend that can be seen is that as more of the intermediate layers are included for training, extending from either the lower layers (left) or higher layers (right), going from the top plots to the lower plots, then the effects become more “mixed” in terms of showing some degree of category-general and category-specific improvements to the output SSNR thresholds that are measured based on classification accuracy.

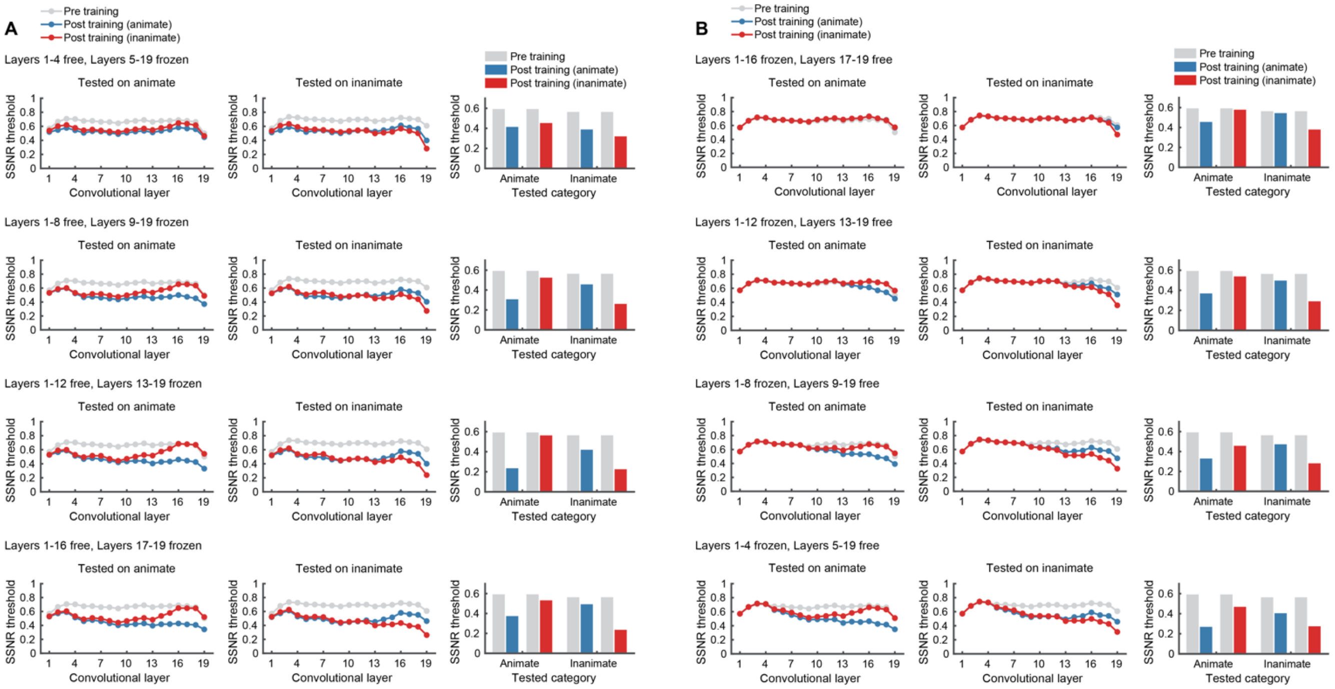

Importantly, these findings do not contradict our original claim that category-general robustness depends more on lower-layer representations while category-specific learning emerges in higher layers. Rather, they suggest that intermediate layers may serve as a bridge, capable of supporting both types of learning depending on how training signals are distributed across the network and which layers are allowed to adapt. Based on the reviewer’s suggestions, we have added the above figure as **Fig S1** to the revised manuscript and briefly discuss the pattern of results found in these supplementary analyses.

3. Regarding my question about individual vs. group-level analyses: I understand that specificities are measured in pre- vs. post-tests, but since these tests are conducted on each subject individually, it should be possible to measure the robustness effect within subjects, in addition to the group-level results currently presented. I'd appreciate clarification on why this individual-level analysis isn't included or feasible.

We thank the reviewer for the follow-up and the opportunity to clarify. To begin, we note that all training effects in the human behavioral data were analyzed based on our measure of SSNR thresholds for each participant, which were then used for within-subject comparisons across pre- and

post-training conditions. As described in the manuscript and shown in **Figs 2B** and **3B**, these individual-level thresholds were analyzed using repeated-measures ANOVAs and paired-sample t-tests, which form the basis of all reported statistical inferences.

If the reviewer was referring to the possibility of analyzing the data of each participant and testing for a statistically significant change in performance within each participant, we note that this type of analysis is not feasible for our experimental design. The SSNR thresholds were estimated using the QUEST adaptive staircase procedure (Watson & Pelli, 1983), a well-established method in psychophysics for estimating an observer's perceptual threshold very quickly with a limited number of trials (in our case, just 48 trials per staircase). Rather than characterizing a highly detailed psychometric function with say 20-30 observations per difficulty level, QUEST adaptively samples different stimulus intensities and computes a threshold estimate based on a Bayesian model to account for the participant's performance across signal strength levels. The amount of data collected from a single QUEST staircase is not enough to test for statistical changes in performance across sessions at the level of the individual observer. Additionally, the test images were randomly assigned to each subject during the pre- and post-training phases, such that participants encountered different images across sessions. This design precludes item-level comparisons within a subject, but was intentional, as we aimed to ensure that observed improvements reflected generalization to novel stimuli rather than image-specific learning effects.
