# Supplementary material for: Category-specific perceptual learning of robust object recognition modelled using deep neural networks
Source: PLoS Comput Biol. 2025 Sep 23;21(9):e1013529. doi: 10.1371/journal.pcbi.1013529 (PMC12478885; doi:10.1371/journal.pcbi.1013529)
Supplement: Attachment — Submitted filename: Jang_NoisePercLearn_RebuttalLetter_2025_09_FT_HJ.pdf [file pcbi.1013529.s007.pdf]

Department of Psychology VANDERBILT College of Arts and Science

September 11, 2025

Dear Dr. Lindsay and Dr. Berry,

Thank you for the careful reading of our manuscript and for the constructive guidance. We are grateful that Reviewers 1 and 3 find their concerns addressed. Regarding Reviewer 2's expressed concerns that our abstract and introductory text implies that perceptual learning is occurring at the superordinate level, we respectfully disagree with this assertion and have made modest changes to the abstract and introduction text so that our experimental design and test category conditions are clearer to the general reader.

First, we would like to point out that the manuscript does not claim superordinate-level (animate vs. inanimate) learning in humans. Throughout, “category-specific” refers to improvements for the categories that were actually trained (i.e., the specific classes shown during noise training), as opposed to improvements that would generalize broadly regardless of what was trained (e.g., general noise filtering). Our intention was not to assert that training produced learning at the superordinate animate/inanimate level, nor to imply transfer to untrained basic-level categories within the same superordinate class.

Second, we believe that the issue of whether learning is occurring at a basic level or superordinate level to lie very far from the main questions of interest in our study. Moreover, we are agnostic to this issue and consider the traditional terminology of “basic-level” and “superordinate-level” to be antiquated, coarse in granularity, and over-simplified. To discuss these concepts early on in the introduction would detract from the main points and narrative flow that we wish to present to the reader, which is why we provide more extensive discussion of these issues in the general discussion.

That said, we are willing to make modest edits to the abstract and introduction to clarify the nature of our experimental design and the nature of the animate and inanimate categories that are trained and tested. For example, in the revised introduction, we now state that “In a second experiment, we evaluated whether these perceptual improvements arose from category-specific or category-general benefits of learning, by training the humans and DNNs on noisy images drawn from the set of 8 animate categories or 8 inanimate categories and subsequently testing with all 16 categories.” This statement makes clear which categories are used for training and which categories are evaluated at test, and indicates to the reader that we will not be testing for generalization to novel categories within the trained superordinate category.

We have also added text to the revised methods, where we state that our behavioral study was not designed to address whether training with a subset of object categories might lead to generalization to novel categories belonging to the trained superordinate category.

We believe the revised manuscript accurately reflects our empirical results and interprets these findings in a clear and balanced manner. If, despite these clarifications and additions, the

manuscript's emphasis is still not considered to be suitable for PLOS Computational Biology, we would prefer to withdraw the manuscript and submit to a venue that will allow us to structure our manuscript according to our preferences.

We thank you for your time and consideration.

Sincerely,

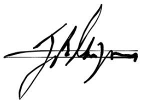

Hojin Jang
Assistant Professor
Korea University

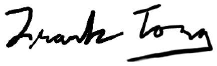

Frank Tong
Centennial Professor of Psychology
Vanderbilt University
